# Intense Focus of Alveolar Echinococcosis, South Kyrgyzstan

**DOI:** 10.3201/eid2406.161641

**Published:** 2018-06

**Authors:** Bakhadyr Bebezov, Nurlan Mamashev, Tilek Umetaliev, Iskender Ziadinov, Philip S. Craig, Deborah E. Joekel, Peter Deplazes, Felix Grimm, Paul R. Torgerson

**Affiliations:** Kyrgyz-Russian Slavic University, Bishkek, Kyrgyzstan (B. Bebezov, N. Mamashev, T. Umetaliev);; University of Zurich, Zurich, Switzerland (I. Ziadinov, D.E. Joekel, P. Deplazes, F. Grimm, P.R. Torgerson);; University of Salford, Greater Manchester, UK (P.S. Craig)

**Keywords:** Kyrgyzstan, Echinococcus multilocularis, alveolar echinococcosis, epidemiology, zoonoses, parasites, parasitic diseases

## Abstract

Human alveolar echinococcosis (AE) is a highly pathogenic zoonotic parasitic disease caused by *Echinococcus multilocularis*. An ultrasound study in southern Kyrgyzstan during 2012 revealed a prevalence of 4.2% probable or confirmed AE and an additional 2.2% possible AE, representing an emerging situation. The risk for probable or confirmed AE was significantly higher in dog owners.

Human alveolar echinococcosis (AE), caused by the larval stage of *Echinococcus multilocularis*, is a lethal parasitic zoonosis if untreated (*1*,*2*). In China, hyperendemic foci of disease have been described (*3*) with prevalences >5%. AE incidence recently has increased in Europe (*4*). In Kyrgyzstan, the disease incidence has increased rapidly since 2000; a total of 148 AE cases were reported in 2013 (*5*).

Hospital records for AE notifications identified a cluster of cases in the Alay Valley in southern Kyrgyzstan. Therefore, in 2012, we conducted an ultrasound study of the population of Sary Mogol (location 39.66°N, 72.88°E) to determine the extent of infection and to investigate the epidemiology of the disease in this district. 

## The Study

The study was a census type of design. We obtained informed consent from each study participant or, for children, consent from parents. Participants were interviewed using a questionnaire in Kyrgyz and given an abdominal ultrasound examination. For participants with hepatic lesions suspected to be AE or cystic echinococcosis (CE) or who reported previous treatment for echinococcosis, a venous blood sample was taken for further analysis. The Ministry of Health of the Kyrgyz Republic provided ethics approval for this study.

We detected specific IgG from collected serum in 3 genus-specific ELISAs based on *E. granulosus* hydatid fluid, native protoscolex antigens, and antigen B (*6*). Specific antibodies against *E. multilocularis* were demonstrated using affinity purified Em2G11 antigen (*6*) and the recombinant Em18 antigen (*7*). We further investigated persons who were negative in these ELISAs with a commercial Western blot (*Echinococcus* western blot IgG; LDBio Diagnostics, Lyon, France).

Where possible, we followed participants to treatment. For some patients we obtained samples from resected lesions. DNA was isolated, followed by amplification of part of the *E. multilocularis* mitochondrial 12S rRNA gene (*8*). Confirmation of diagnosis was also achieved by histologic examination of the resected lesions. Possible AE cases were those with ultrasound lesions and no follow-up. Probable cases additionally had positive serologic results, and confirmed cases were positive by histology, PCR, or both.

We analyzed data with all AE cases and with probable or confirmed AE as the dependent variable using a relative risk generalized linear model (GLM). We analyzed differences in lesion sizes between seropositive and seronegative groups by the Wilcoxon test and used Fisher exact test to examine differences in seroprevalence between persons with confirmed AE and persons with only an ultrasound diagnosis.

**Figure 1:**
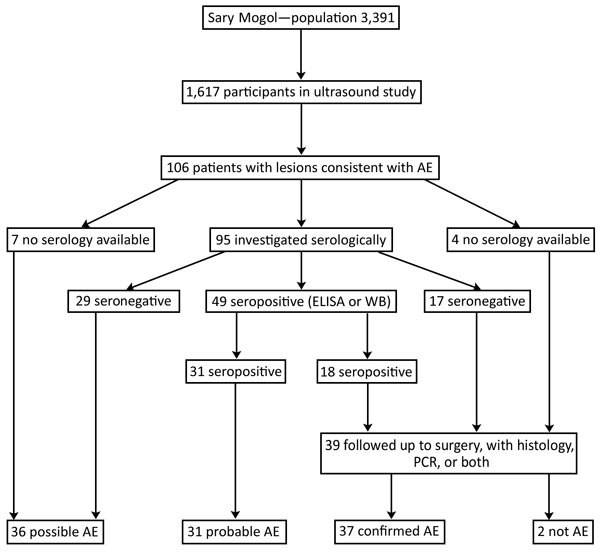
Flowchart of patient selection, ultrasound investigation, serologic testing, and case definitions in a study of AE, southern Kyrgyzstan, 2012. AE, alveolar echinococcosis; WB, Western blot.

We examined 1,617 persons (Figure 1) (48% of the population of the district; Technical Appendix). Of these, 106 persons had ultrasound findings consistent with AE (including 1 concomitantly infected with CE). Probable or confirmed cases (online Technical Appendix) with >2 diagnostic criteria (Figure 1) were subsequently diagnosed in 68 (4.2%) person leaving 36 (2.2%) with possible AE. Three (0.2%) additional persons had lesions suggestive of CE. For 9 persons, images were recorded as inconclusive.

The GLM demonstrated an increase in the risk for possible infection with AE in dog owners, male patients, and persons who practiced home slaughter of livestock. Only dog ownership increased the risk for probable AE infection (Table 1).

**Table 1:** Relative risk from multivariable analysis of persons with an ultrasound diagnosis of AE and persons with a probable or a confirmed diagnosis of AE, southern Kyrgyzstan, 2012*

Dependent variable, risk factor	Relative risk (95% CI)	p value
Ultrasound diagnosis of AE		
Patient age†	0.982 (0.969–0.995)	0.0074
Male sex	1.56 (1.07–2.29)	0.021
Dog ownership	1.82 (1.24–2.72)	0.0025
Home slaughter of livestock	1.60 (1.03–2.56)	0.043
Dog ownership among persons with probable and confirmed AE‡	2.81 (1.64–5.09)	0.00033

*Probable diagnosis: ultrasound and serology; confirmed diagnosis: ultrasound and histology/PCR. AE, alveolar echinococcosis. †Median age of persons with possible AE was 24 y; median age of AE-negative persons was 28 y. ‡Dog ownership was the only risk factor remaining as significant when only probable and confirmed AE were analyzed as the dependent variables.

**Figure 2:**
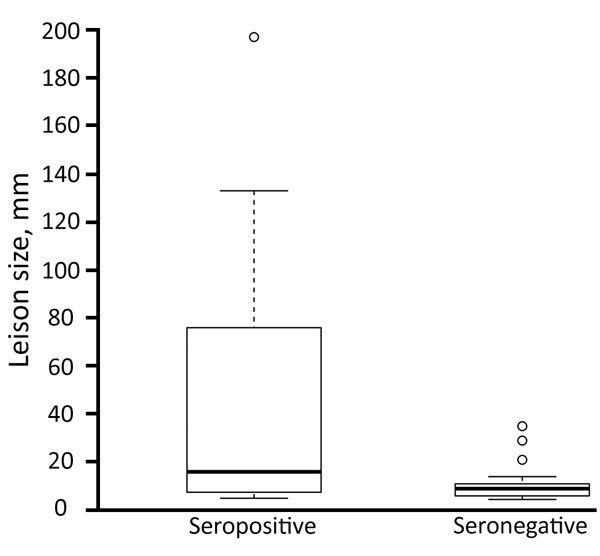
Differences in sizes of lesions (n = 49 serum samples available from 57 patients with measured lesions) diagnosed in persons seropositive by ELISA, Western blot, or both (n = 27) or seronegative (n = 22) in a study of alveolar echinococcosis, southern Kyrgyzstan, 2012. Box plots indicate interquartile range (box top and bottom), median (black horizontal line), 1.5 times interquartile range (error bars), and extreme values (circles).

Of the 106 persons in whom AE was diagnosed, we detected specific antibodies in 40 (42.1%) of the 95 available serum samples by 3 different ELISAs (Table 2). Western blot analysis of negative serum identified specific antibodies on *Echinococcus* genus level in 9 additional patients. Thus, 49 of the 95 persons had serologic evidence of infection. Lesions, measured in 53 patients, ranged from 5 to 197 mm (mean 28 mm). The mean size of lesions in the 22 ELISA- or Western blot–seropositive persons was 46.1 mm, significantly larger than the mean size of 11.0 mm for lesions from the 27 seronegative patients (p = 0.01; Figure 2).

**Table 2:** Ultrasound results partially confirmed with PCR/histology for AE or CE in relation to serology, southern Kyrgyzstan, 2012*

Ultrasound results	ELISA					WB†				
	No. available samples‡	Neg	AE/CE§	AE¶		No. available samples‡	Neg	AE/CE	CE	AE
AE, n = 106	95	55	25	15		43	34	6	3	0
Confirmed AE,# n = 37	33	18	10	5		13	10	3	0	0
Inconclusive, n = 9	6	5	1	0		4	4	0	0	0
CE, n = 3	3	2	1	0		0	0	0	0	0
CE and AE, n = 1	1	0	1	0		0	0	0	0	0
Negative but with history of CE/AE, n = 13	6	1	3	2		1	1	0	0	0

*AE/CE indicates the test is specific only to genus level. AE, alveolar echinococcosis; CE, cystic echinococcosis; neg, negative; WB, Western blot. †ELISA-negative serum only. ‡Not every patient for whom echinococcosis was diagnosed by ultrasound had inconclusive results or a history of echinococcosis provided a blood sample. For some of these patients, insufficient serum was available from the blood sample to undertake both ELISA and WB. §*E. granulosus* hydatid fluid and/or native protoscolex antigens and/or antigen B positive but Em18 and EmG11 negative. ¶Em18 and/or EmG11 positive (*E. granulosus* hydatid fluid and/or native protoscolex antigens and/or antigen B positive or negative). #Diagnosis confirmed by PCR and/or histology of resected lesion.

By September 2017, a total of 39 persons were known to have been treated by hepatic surgery. Among them, AE was confirmed in 37 (94.9%) by histology, PCR, or both. From these 37 persons, 35 serum samples were available; 18 (51.4%) showed serologic evidence of infection. This finding did not differ significantly from the proportion of persons without follow-up data who had serologic evidence of infection (31/61) (Figure 1).

The decreasing risk for possible AE with increasing age, contrasting with findings in areas of China where AE is highly endemic (*9*), indicates different dynamics and hence reflect an emerging epidemic of human AE in Kyrgyzstan. Consequently, this observation supports the hypothesis that the epidemic could be linked to the dissolution of the Soviet Union in 1991 (*5*). In our study, the higher risk for possible AE in male than in female patients contrasts with risk in areas of western China where AE is endemic. The reasons for this difference are unclear but might reflect behavioral (e.g., rates of dog contact) or cultural reasons that result in a greater risk for exposure for female persons in China (*9*) and for male persons in Kyrgyzstan. However, both risk factors disappear if only probable or confirmed AE is used as the case definition for AE.

The poorer sensitivity (≈50%–60%) of the serologic tests as compared with the validations in Switzerland (*6*) might result from cases in Switzerland being at a more advanced stage of clinical disease. The fact that persons with larger lesions were more likely to be seropositive indicates that seroconversion might not occur either during the early stages of the disease or when only abortive lesions are present. Similar patterns of low seroreactivity were observed in the AE endemic focus in south Gansu (China) (*10*) and included persons with possible abortive forms of the disease. In addition, the mean age of ultrasound-positive persons in our study is 9 years younger than those receiving surgical treatment resulting from clinical disease, indicating that our study has detected an early stage of the disease in these persons. In the patients followed up, the seropositivity rate for those with AE confirmed by histopathology did not differ significantly from the rate for those with only an ultrasound diagnosis. Thus, we can conclude that the same proportion of patients without histologic or PCR confirmation (to date) are likely to have AE. Although the diagnostic efficiency of ultrasound should be estimated with caution, these results might indicate a specificity as high as 99.7% (online Technical Appendix). However, including only probable or confirmed cases in the regression analysis increased the association with dog ownership while eliminating other risk factors. This finding might indicate that some of the possible AE cases are not AE. Nevertheless, specificity of ultrasound in this scenario remains at 97.4%.

## Conclusions

We documented a highly endemic focus of human AE in which the prevalence of confirmed or probable AE was ≈4.2% in southern Kyrgyzstan. A characteristic of communities with high levels of human AE are concomitant high prevalences of *E. multilocularis* in the dog population, such as western Sichuan Province (*11*). The mole vole (*Ellobius tancrei*) has recently been confirmed as a natural intermediate host of *E. multilocularis* in Sary Mogol and has identical DNA sequence for the *E. multilocularis* haplotype described in feces of local domestic dogs (*12*). In Kyrgyzstan, prevalences in dogs of 20% have been observed (*13*). In our study, dog owners had 1.8 times higher risk for infection than non–dog owners, increasing to 3.3 times for confirmed or probable infection, thus providing evidence that dogs are involved in transmission to humans.

Technical Appendix Study population, details of generalized linear model, patient details, and accuracy of diagnostic procedures in a study of alveolar echinococcosis, southern Kyrgyzstan, 2012.
