# Supplementary material for: Intense Focus of Alveolar Echinococcosis, South Kyrgyzstan
Source: Emerg Infect Dis. 2018 Jun;24(6):1119–22. doi: 10.3201/eid2406.161641 (PMC6004877; doi:10.3201/eid2406.161641)
Supplement: Technical Appendix — Study population, details of generalized linear model, patient details, and accuracy of diagnostic procedures in a study of alveolar echinococcosis, southern Kyrgyzstan, 2012. [file 16-1641-Techapp-s1.pdf]

Article DOI: https://doi.org/10.3201/eid2406.161641

# Intense Focus of Alveolar Echinococcosis, South Kyrgyzstan

## Technical Appendix

## Study Population

This study was conducted in the community of Sary Mogul in southern Kyrgyzstan (Technical Appendix Figure 1). The expected age distribution if ultrasound participants were precisely representative of census data are in Technical Appendix Table 1. The actual proportions by age and sex is in Technical Appendix Table 2. The differences between the expected proportions and actual population investigated is significant ($p<0.0001$, $\chi^2$ test) Thus, there is an overrepresentation of adult females in the ultrasound surveillance group compared to the expected population according to the census. This may be due to men working (either locally or even abroad—a large number of Kyrgyz men of working age work in Russia ([*1*]) and send remittances back to Kyrgyzstan). Thus, they may not have been available. There was also some underrepresentation of very young children <4 years) and underrepresentation of elderly persons >70 years.

## Details of Generalized Linear Model

Variables examined in the generalized linear model (GLM): Age, sex, size of household, length of time resident in Sary Mogul, occupation, dog ownership: if yes—type of dog (hunting or other), dog allowed to roam; visit to summer pastures, living standard, disposal of dog feces, children playing with dog, dog treated with antihelmintics, water supply (stream, well, pipe), wash fruit and vegetables, wash hands before eating, slaughter livestock, feed infected organs from slaughtered livestock to dogs, know about echinococcosis, previous infection with echinococcosis.

The GLM was a binomial model with a log link with ultrasound positive for alveolar echinococcosis (AE) as the dependent variable. The log link (rather than logit) gives an

interpretation in terms of significant variable as relative risk of infection associated with that variable (as opposed to the logit, which gives the odds ratio). All computations were undertaken in R (https://www.r-project.org). A backward selection method was used with all variables initially included; with each iteration, the least significant variable was removed until only variables with $p<0.15$ remained in the model. Variables with $p<0.05$ in the final model were reported as significantly associated with the presence of an AE lesion diagnosed by ultrasound.

The adequacy of the model was addressed by a sensitivity analysis using R. Briefly the data were repeatedly and randomly split in subsets to address the predictive value of the model. Thus, parameters were derived from part of the data and used to predict the accuracy of the model on the remainder of the data. From these, an estimate of the sensitivity and specificity of the model can be made, as well as the overall accuracy (area under the curve in a Receiver Operating Characteristic Curve plot). The Area Under the Curve was 0.64 for the binomial model, which indicates the model had a modest predictive power, despite the significant parameters (Technical Appendix Figure 2). In addition the pseudo$R^2$ was 0.046 indicating that the final model parameters: dog ownership, sex, age and slaughter of livestock could only predict ≈4.6% of the variation in infection with AE. Variable importance analysis indicated that dog ownership (3.02) had the greatest influence, followed by age of participant (2.68), sex (2.30), and home slaughter (2.02).

## Patient Details

Of the 1,617 study participants examined by ultrasound, suspected AE was diagnosed in 106; in 1, cystic echinococcosis (CE) was concomitantly diagnosed. CE was diagnosed in 3 additional participants. Of these, 53 patients with AE had the lesions measured. The mean age of those with measured lesions was 28 years, and 49% were male; mean of those with unmeasured lesions was 20 years and 61% were male. Of the 37 patients who had follow up and lesions confirmed as AE by histopathology, 13 had been measured at the initial ultrasound scan. These had a mean size of 40.1 mm (range 7–133 mm). Of the remaining 70 who were lost to follow up, 40 had their lesions measured during the original ultrasound scan and had a mean lesion size of 25.5 mm (range 5–197 mm). There was some statistical evidence that the group with follow up had larger lesions (Wilcoxen test, $p = 0.02$).

CE was diagnosed in only 3 participants (a prevalence of just 0.2%), which is too small a sample size to undertake any analysis for this disease. It is also a somewhat lower prevalence than found in similar studies elsewhere in Kyrgyzstan (*2*) or in a neighboring region of Kazakhstan (*3*). The reasons for this are not known, but ongoing research mapping all cases of echinococcosis and analyzing the geographic distribution may give clues to this.

## Accuracy of the Diagnostic Procedures

Diagnostic accuracy of the serology and ultrasound examination can be assessed only against proven AE infections. We have examined 3 possible scenarios, all with the assumption that ultrasound has a diagnostic sensitivity of 100% (Technical Appendix Table 3):

1. Of the 39 patients followed up through treatment, 37 were proven to have AE after histopathology and/or PCR of the resected lesion. Of these 37 patients, 33 were examined serologically (4 serum samples were not available). Of the 33 serum samples, 18 had serologic evidence of infection through ELISA or Western blot, indicating sensitivity of serology to be 18/33 or 54.5% (exact binomial 95% CI 36.4%–71.9%). Thus, there are substantial numbers of false negatives suggesting that serology on its own is an insensitive diagnostic technique for this population. It is not possible to estimate the specificity of the serology without serologic data from those patients who were ultrasound negative, and serum samples were not taken for such serologic investigation. For the ultrasound examination, of 39 patients with a diagnosis of AE on ultrasound, 37 patients were proven to have AE by histopathology after lesion resection. If these 37 patients are assumed to be representative of the 106 patients who had a ultrasound diagnosis of AE, then it would be expected that ≈2/39 × 106 would be false positives = 5 false positives. Thus, 101 of the 106 would be true positives. Therefore, of the 1,617 patients examined, 1,516 would not be infected with AE. The ultrasound would have correctly identified 1,516 – 5 = 1,511 as not infected. Thus, the specificity of ultrasound can be estimated to be 1,511/1,516 = 99.7%. The prevalence of infection, assuming this specificity would be 101/1,617 = 6.2%.

2. A lower estimate of the specificity could be made by assuming that the 67 cases without follow up were all AE negative; thus, the false positives would be 69 of the 106 cases. In this instance, the number of patients not infected with AE would be 1,617–37 = 1,580, and the ultrasound correctly diagnoses 1,511 as not infected, giving a specificity of 1,511/1,580 = 95.6%.

The prevalence of infection, assuming this lower specificity, would be 37/1,617 = 2.3%. These all assume that the sensitivity of ultrasound is near 100%. This is reasonable because a case definition is a visible lesion consistent with AE on ultrasound. It is possible that there may have been cases of AE without a primary lesion in the liver, which would have escaped detection by ultrasound, but such cases are rare. Just 9 of 387 AE cases in France had primary extrahepatic lesions (*4*).

3. Alternatively 49 study participants (of 95 with serology) were serologically positive, and so probably had AE. In addition, there were 18 confirmed cases of AE (seronegative or no serum sample, but confirmed by histology), which would suggest 67 cases as confirmed or probable AE. This gives a prevalence of 4.1% and a specificity of the ultrasound as 97.4% (39 false positives in 1,550 not infected)

The sensitivity of the serology varies little between scenario 1 and 2, and we cannot estimate it in scenario 3. The positive predictive values (i.e., the probability of having AE given a positive diagnosis with ultrasound) varies from 34.9% to 94.8% depending on which scenario is adopted. The prevalence also varies, but even with the most conservative estimate, assuming all cases lost to follow up were AE negative, it still gives a high prevalence of 2.3%.

**References**

1. OECD iLibrary. International migration outlook 2013 [cited 2017 Oct 1]. http://www.oecd-ilibrary.org/social-issues-migration-health/international-migration-outlook-2013_migr_outlook-2013-en

2. Torgerson PR, Karaeva RR, Corkeri N, Abdyjaparov TA, Kuttubaev OT, Shaikenov BS. Human cystic echinococcosis in Kyrgystan: an epidemiological study. Acta Trop. 2003;85:51–61. PubMed http://dx.doi.org/10.1016/S0001-706X(02)00257-7

3. Torgerson PR, Rosenheim K, Tanner I, Ziadinov I, Grimm F, Brunner M, et al. Echinococcosis, toxocarosis and toxoplasmosis screening in a rural community in eastern Kazakhstan. Trop Med Int Health. 2009;14:341–8. PubMed http://dx.doi.org/10.1111/j.1365-3156.2009.02229.x

4. Piarroux M, Piarroux R, Giorgi R, Knapp J, Bardonnet K, Sudre B, et al. Clinical features and evolution of alveolar echinococcosis in France from 1982 to 2007: results of a survey in 387 patients. J Hepatol. 2011;55:1025–33. PubMed http://dx.doi.org/10.1016/j.jhep.2011.02.018

**Technical Appendix Table 1.** Expected age and sex distribution of the 1,617 participants of the ultrasound surveillance based on the Kyrgyz census data*

| Age, y | Female | Male | % Female |
|---|---|---|---|
| 0–4 | 102.706499 | 106.896314 | 0.0635167 |
| 5–9 | 92.5792435 | 88.1877789 | 0.05725371 |
| 10–14 | 93.4082444 | 90.9436469 | 0.05776638 |
| 15–19 | 84.8493695 | 89.8457808 | 0.05247333 |
| 20–24 | 77.7020369 | 81.8918526 | 0.04805321 |
| 25–29 | 68.4485936 | 69.5240543 | 0.04233061 |
| 30–34 | 55.0501455 | 60.1585839 | 0.03404462 |
| 35–39 | 45.6174588 | 51.3980601 | 0.02821117 |
| 40–44 | 42.3910766 | 41.6741028 | 0.02621588 |
| 45–49 | 40.3521823 | 36.7449079 | 0.02495497 |
| 50–54 | 29.8888458 | 27.357032 | 0.01848413 |
| 55–59 | 22.0021339 | 18.1259942 | 0.01360676 |
| 60–69 | 22.786324 | 20.7698351 | 0.01409173 |
| ≥70 | 30.6506305 | 25.0492726 | 0.01895524 |
| Total | 808.432784 | 808.567216 | 0.4997 |

*The total population of Sary Mogul, Kyrgyzstan, is 3,391.

**Technical Appendix Table 2.** Actual age and sex distribution of the 1,617 participants of the ultrasound surveillance of alveolar echinococcosis, southern Kyrgyzstan, 2012

| Age, y | Female | Male | % Female |
|---|---|---|---|
| 0–4 | 25 | 33 | 0.01546073 |
| 5–9 | 104 | 89 | 0.06431664 |
| 10–14 | 111 | 112 | 0.06864564 |
| 15–19 | 94 | 57 | 0.05813234 |
| 20–24 | 62 | 39 | 0.03834261 |
| 25–29 | 96 | 68 | 0.0593692 |
| 30–34 | 97 | 64 | 0.05998763 |
| 35–39 | 85 | 40 | 0.05256648 |
| 40–44 | 70 | 38 | 0.04329004 |
| 45–49 | 58 | 21 | 0.03586889 |
| 50–54 | 69 | 31 | 0.04267161 |
| 55–59 | 51 | 22 | 0.03153989 |
| 60–69 | 32 | 15 | 0.01978973 |
| ≥70 | 16 | 8 | 0.00989487 |
| Total | 970 | 637 | 0.5999 |

**Technical Appendix Table 3.** Sensitivity and specificity of ultrasound and serology under varying assumptions in a study of alveolar echinococcosis, southern Kyrgyzstan, 2012

| | Ultrasound | | | | |
|---|---|---|---|---|---|
| Assumption | Sensitivity, % | Specificity, % | Positive predictive value, % | Serology, sensitivity, % | Prevalence, % |
| Confirmed cases are representative | 100 | 99.7 | 94.8 | 54.5 | 6.2 |
| Nonconfirmed cases are negative | 100 | 95.6 | 34.9 | 51.4 | 2.3 |
| Confirmed and/or seropositive cases are probable alveolar echinococcosis | 100 | 97.4% | 63.2 | | 4.1 |

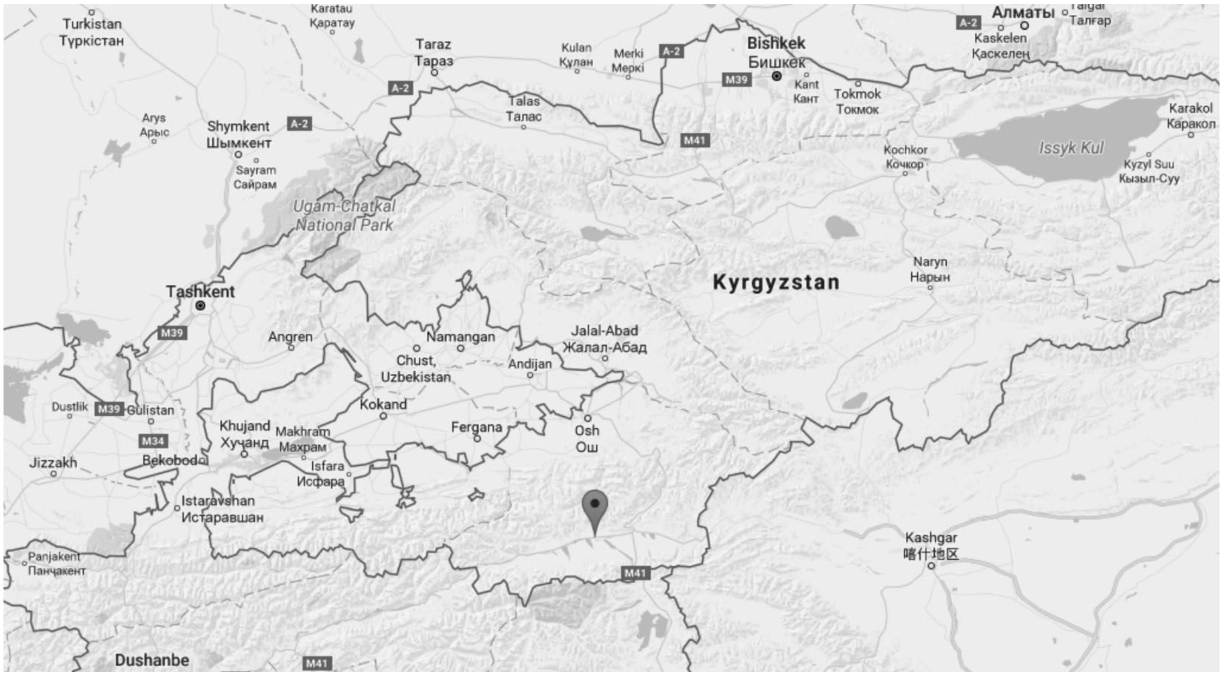


**Technical Appendix Figure 1.** Location of study area, Sary Mogul, southern Kyrgyzstan. Map data © 2017 Google.

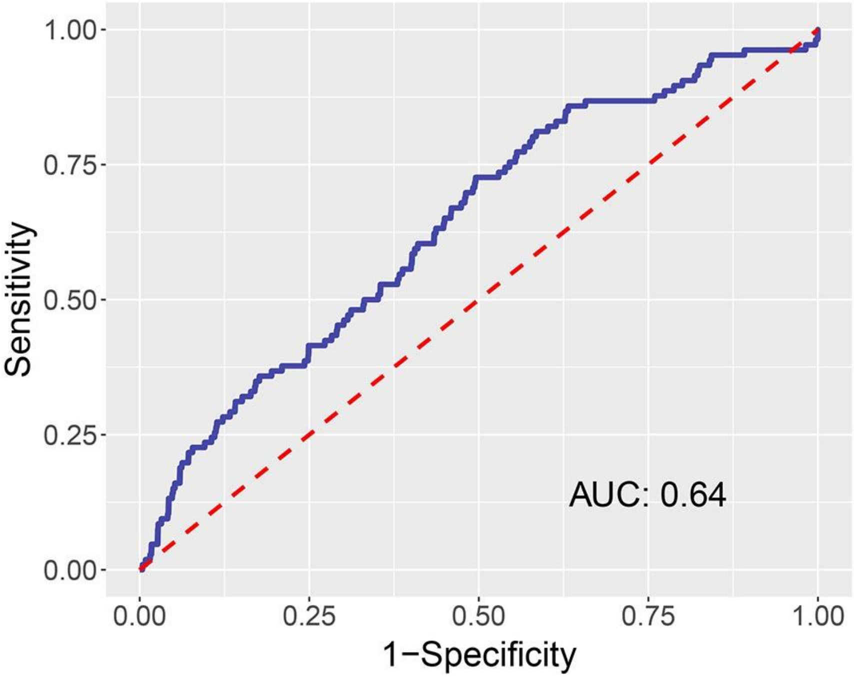


**Technical Appendix Figure 2.** Analysis of relative risk regression results indicating the sensitivity and specificity of the predictions given by the model. Purple line indicates Receiver Operating Characteristic Curve; red line indicates line of no-discrimination.
